# Computer and robotic – assisted total knee arthroplasty: a review of outcomes

**DOI:** 10.1186/s40634-020-00278-y

**Published:** 2020-09-24

**Authors:** Jobe Shatrov, David Parker

**Affiliations:** 1 Royal North Shore Hospital, St Leonards, Australia; 2 Sydney Orthopaedic Research Institute, Chatswood, Australia; 3 University of Sydney, Sydney, Australia

## Abstract

**Background:**

Total knee arthroplasty (TKA) is a successful treatment for tricompartmental knee arthritis. Computer navigation and robotic-assisted-surgery (RAS) have emerged as tools that aim to help plan and execute surgery with greater precision and consistency. We reviewed the most current literature to describe the historical background and outcomes compared to conventional TKA.

**Methods:**

A review and synthesis of the literature comparing the patient reported outcomes (PROM’s) of RA TKA and computer-assisted (CA) TKA to conventional TKA was performed using the Preferred Reporting Items for Systematic Reviews and Meta-Analyses (PRISMA) guidelines.

**Results:**

CAS TKA improves accuracy and consistency of implant position, and appears to provide a small improvement in PROMs and implant survival compared to conventional TKA. RTKA similarly improves implant accuracy compared to conventional techniques and early results suggest a similar small benefit in PROMs compared to conventional TKA. A strengthening trend is emerging showing CAS TKA has greatest benefit to implant survival in people under 65. RTKA survival analysis data is more limited and early results do not allow strong conclusions, however early trends are similar to CAS TKA.

**Conclusion:**

Results for CAS-TKA show improvement in alignment, and early clinical outcomes have revealed promising results, with longer-term data and medium-term survival analysis recently emerging showing small benefits over conventional TKA. RTKA represents another phase of development. Early results show similar trends to that of CAS TKA with longer-term data still to come.

## Introduction

Total knee arthroplasty (TKA) is a successful treatment for tricompartmental knee arthritis. Emphasis on optimal component sizing and alignment has led to increased use of tools that allow the delivery of pre-operative plans and verification of intra-operative steps. Computer navigation and robotic-assisted-surgery (RAS) have emerged as tools that aim to help plan and execute surgery with greater precision and consistency, with the ultimate goal of improving patient outcomes in TKA.

Computer-assisted (CAS), or navigation-assisted refers to a device that has an interface that allows entry of anatomical data, and then gives feedback to a surgeon regarding alignment of implants and overall alignment of the knee, but cannot be programmed to perform tasks. Multiple proprietary systems now exist and rapid technological advancements in computer processing power have stimulated development of robotic surgical systems. Robotic systems generally provide similar feedback to CAS systems, but can also be programmed to assist in the execution of certain surgical tasks.

Emerging data on the use of this technology in uni-compartmental knee replacement (UKR) suggests an improvement in outcomes and survival at 2 years (2.8 vs 4.6%) compared to conventional techniques [1, 2], with short-term results showing similar outcomes for both CAS and robotically assisted UKR [3]. There remains conflicting evidence as to whether increasing use of CAS and RAS results in either improved survivorship or patient reported outcomes measures (PROMs) following TKA.

The purpose of this review is to describe the historical background of robotic and computer-navigated systems used most commonly for total knee replacements, and review the most current available literature regarding outcomes compared to conventional TKA.

## Methods

A review and synthesis of the literature comparing the PROMs of robotic-assisted total knee arthroplasty (RATKA) and CAS TKA to conventional TKA was performed using the Preferred Reporting Items for Systematic Reviews and Meta-Analyses (PRISMA) guidelines [4]. Primary outcome of interest was survivorship, with secondary outcome measures being PROMs.

### Search strategy

The online databases Pubmed, Embase and CENTRAL (Cochrane Central Register of Controlled Trials) were searched. Publicly available registry data was also searched.

A search was performed on the 3.4.2020 using combined text and MESH terms: “Robotic Arm-assisted total knee arthroplasty”, “robotic assisted total knee arthroplasty”, “robotic knee arthroplasty”, “robotic-assisted primary total knee arthroplasty,” “computer assisted total knee arthroplasty”, “computer assisted knee arthroplasty” and “computer assisted primary knee arthroplasty”.

### Study selection and screening

A total of 3157 abstracts were identified for further screening in two-stages. Abstracts were screened for data that compared PROMs or survivorship analysis of either CAS TKA or RATKA to conventional TKA. Full-text was downloaded and the article further assessed for eligibility based on inclusion criteria. Reference lists of selected articles were also searched for any additional articles.

### Computer-assisted total knee Arthroplasty

A total of 2652 abstracts were identified from the initial search. After first stage screening a total of 135 full texts were assessed based on the listed eligibility criteria. 31 studies were found to be suitable for analysis in the final review of CAS TKA versus conventional TKA.

### Robotic-assisted total knee Arthroplasty

A total of 705 abstracts were identified for further screening. After first stage screening a total of 34 full texts were assessed based on the listed eligibility criteria. 13 studies were found to be suitable for analysis in the final review of CAS TKA versus conventional TKA.

### Eligibility criteria

Articles comparing outcomes of CAS TKA or RTKA to conventional TKA, published after the year 2000, adequate definition of robotic or computer assisted arthroplasty were included. Articles were excluded if they were not published in English, if data regarding survivorship, radiographic outliers or PROM’s were not extractable from the results, operation after than total knee replacement performed, case reports or were duplicate studies (eg. publishing 5 year data with earlier article publishing 2 year data). Only level 1 to 3 studies according to AAOS grades of evidence were included in this review [5].

For the CAS TKA analysis only articles with minimum two-year follow-up were accepted. For RATKA, data at any follow-up period was accepted due to the published data in this area being relatively new.

### Historical perspective

#### Computer-assisted TKA

The first navigated total knee replacement was performed in Grenoble in 1997 using an image-free navigation system [6], which used a kinematic model to determine the mechanical alignment of the limb. Later systems added anatomic landmarks from the knee and ankle to improve accuracy.

**Fig. 1:**
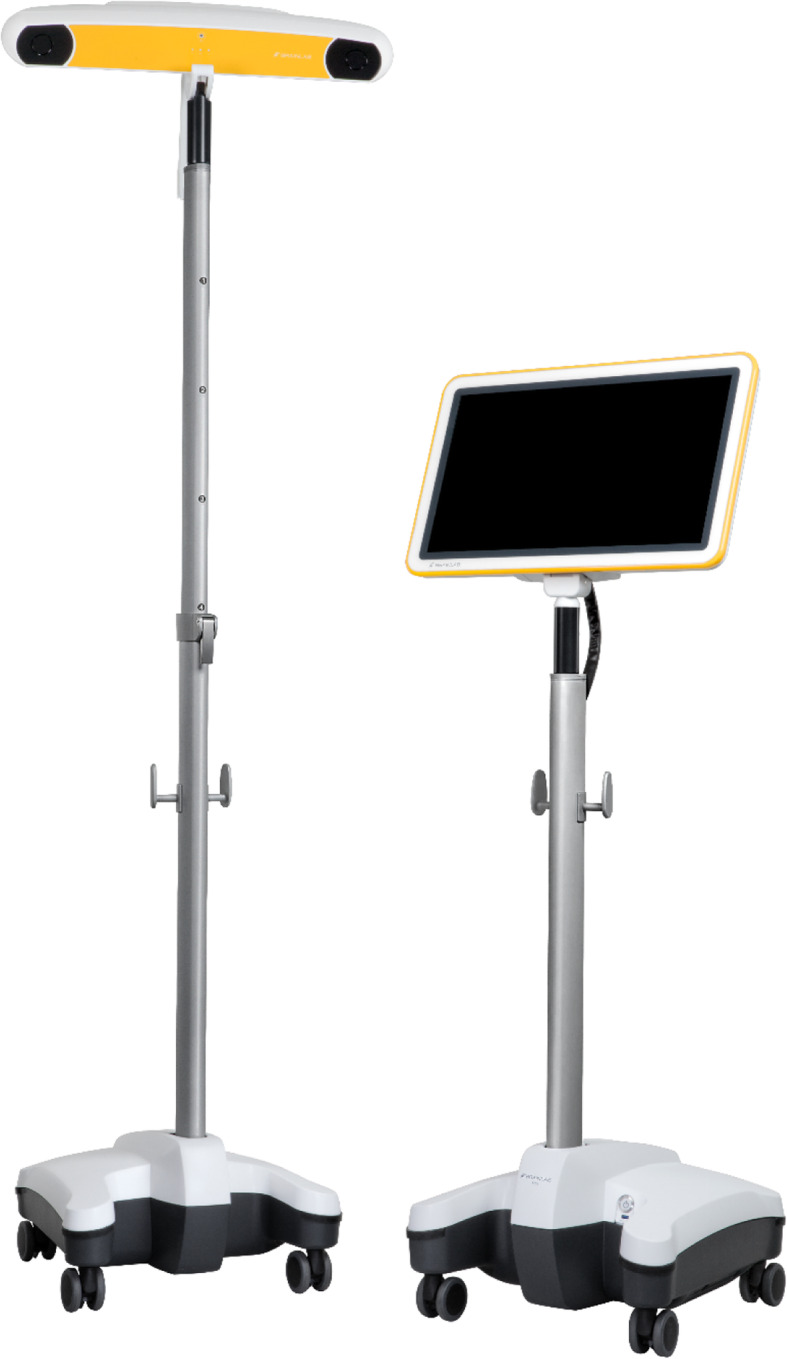
BrainLab navigation unit illustrating motion capture camera and computer screen interface

Most systems currently operate by use of cameras that allow entering of anatomical data via infra-red signal, and this data is then used to analyse anatomical morphology, alignment, movement and surgical instrument position (see example Fig. 1). The system will often provide a suggested plan to the surgeon, which can be over-ridden at any point. Most systems allow cuts to be verified and measured to check for any deviation from the surgical plan, however this step is not mandatory, allowing for significant deviations from the planned cuts to be made without being measured or reported in the literature.

CAS TKA has evolved to now have 2 main categories, image-based and image-free. Initial systems were either based on fluoroscopic images or an imageless CAS navigation system that required intraoperative registration of the hip and ankle center, joint surface, and various other landmarks around the knee, to create a virtual co-ordinate system that guides resection according to the desired alignment. Image-based systems were later developed using pre-operative CT and MRI to provide registration of the joint surface and overall alignment. In some instances, these systems have had additional customised cutting jigs, or ‘patient specific guides’ created to be used in conjunction with the CAS. More recently, accelerometer based hand-held navigation systems have been developed to assess alignment and tool position without the need for large console monitors or computer platforms. Image-based systems have recently grown in popularity with the emergence of RATKA.

Several navigations systems exist for CAS or navigated TKA. The most common are, Stryker CT-free navigation (Navigation System II; Stryker, Mahwah, New Jersey), OrthoPilot CT-free navigation (OrthoPilot version 4.2; B. Braun Aesculap, Tuttlingen, Germany), and VectorVision CT-based navigation (VectorVision version 1.6; BrainLAB, Munich, Germany) [7]. An important difference to robotic systems is that navigation units can be used with a variety of prostheses. Furthermore, differences exist between navigation systems: for example, Stryker navigation can either be an articular surface mounted (ASM) system or the precision navigation system (now called OrthoMap). In the former, the system assists with just the distal femoral and proximal tibial cuts, with no subsequent feedback regarding overall alignment or balancing. The latter also assists with these cuts, which can be verified, but also assists with implant sizing and positioning and gives feedback on alignment and balancing. Of note is that these important differences are often not controlled for in most studies assessing computer assistance with TKR.

#### Robotic Total knee Arthroplasty

Two early robotic systems were developed in the 1980’s for use in knee arthroplasty. The first robotic TKA was performed in 1988 using the ACROBOT (Active Constraint Robot) robotic system (Imperial College, London, United Kingdom) [8]. The company withdrew from robotics and MAKO Surgical acquired the business as part of a confidential patent infringement settlement in 2013 [9]. The CASPAR system (URS Ortho Rastatt, Germany) was also an image-guided active robot used for total hip arthroplasty and TKA. The first TKA performed was in March 2000 in Germany at the Kassel Orthopaedic clinic as part of a prospective trial after earlier being tested on saw bones and cadaveric models [10]. The CASPAR robotic system is no longer available for clinical use.

As computer-assisted surgery became more popular, several more robotic systems were developed. The ROBODOC system (Initially by Curexo Technology, Fremont, Ca, now called Think Surgical, Inc. in September, 2014) was developed and became the first robot to be used in orthopaedic surgery in the clinical setting. Initially designed for use in hip arthroplasty, a platform was later developed for use in TKA. Most of the early data regarding outcomes of robotically-assisted TKA are with the use of the ROBODOC robotic platform. In South Korea ROBODOC was first used in the clinical setting in 2001, and by 2007 more than 2000 TKA’s had been performed using this system [11]. The Omnibotic robot (previously Praxim) was later approved for clinical use in 2010 and is used exclusively for TKA. Like all current robotic systems currently available for clinical use, early published data showed improved accuracy of cuts compared to conventional TKA in cadaveric models [12]. Omnibotics was acquired by CORIN group in March 2019.

The Robotic Arm Interactive Orthopedic System (RIO; MAKO Stryker, Fort Lauderdale,Florida), which utilises haptics, was more recently approved for commercial use in TKA, and has been the focus of most of the recently published literature regarding robotically-assisted TKA outcomes. Some early data has suggested that this system causes less soft tissue trauma and post-operative pain resulting in earlier discharge when compared to conventional techniques [13, 14]. The Rosa Knee System (Zimmer Biomet, Warsaw, Indiana) is the latest robotic system approved by the FDA in January 2019. These later robotic systems have been validated in cadaveric models to show highly accurate bone cuts to achieve planned angles and resection thickness to less than 1 mm error [15].

**Fig. 2:**
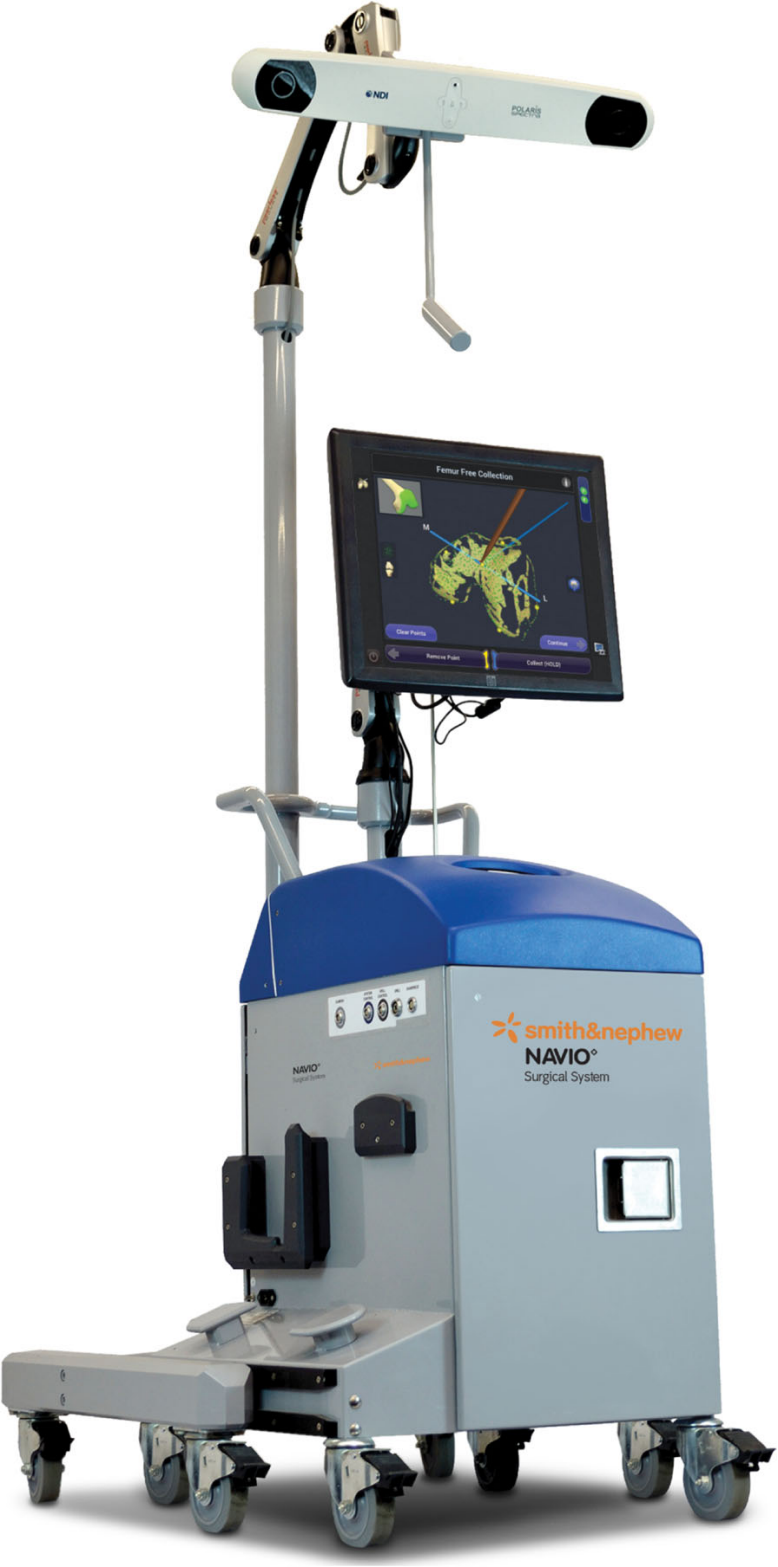
NAVIO robotic system (Smith and Nephew) demonstrating the robotic unit with computer interface for surgeon feedback and motion capturing camera

**Fig. 3:**
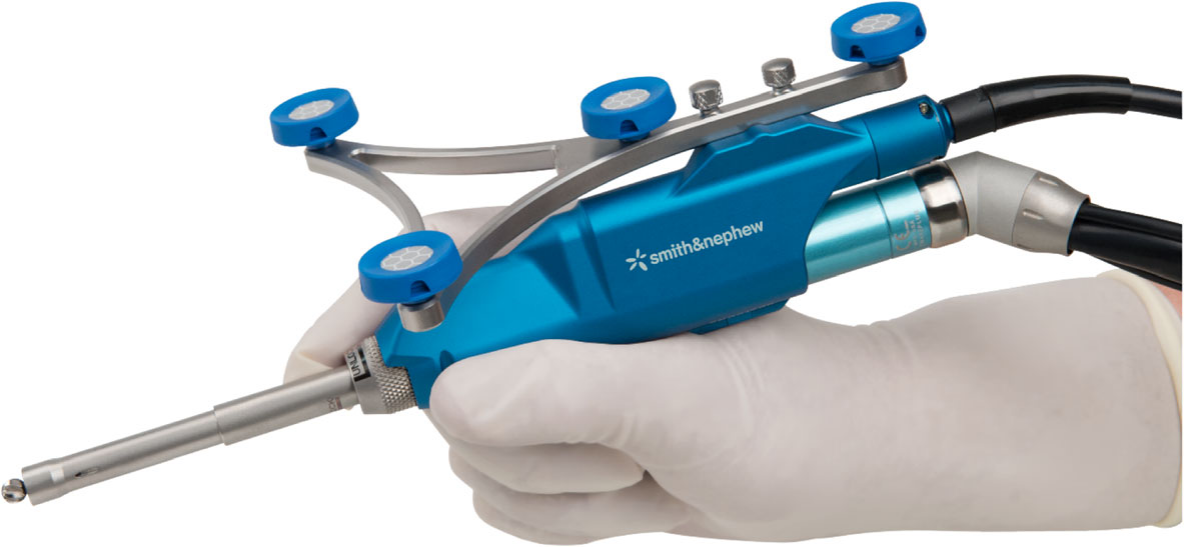
Cutting burr connected to the robotic unit in image 2. The handpiece delivers the surgical plan determined by the surgeon. Whilst the surgeon still has some control over the movement of the cutting tool, some robotic systems are designed to ‘cut-out’ when the instrument strays from planned surgical resection margins on the plan

**Fig. 4:**
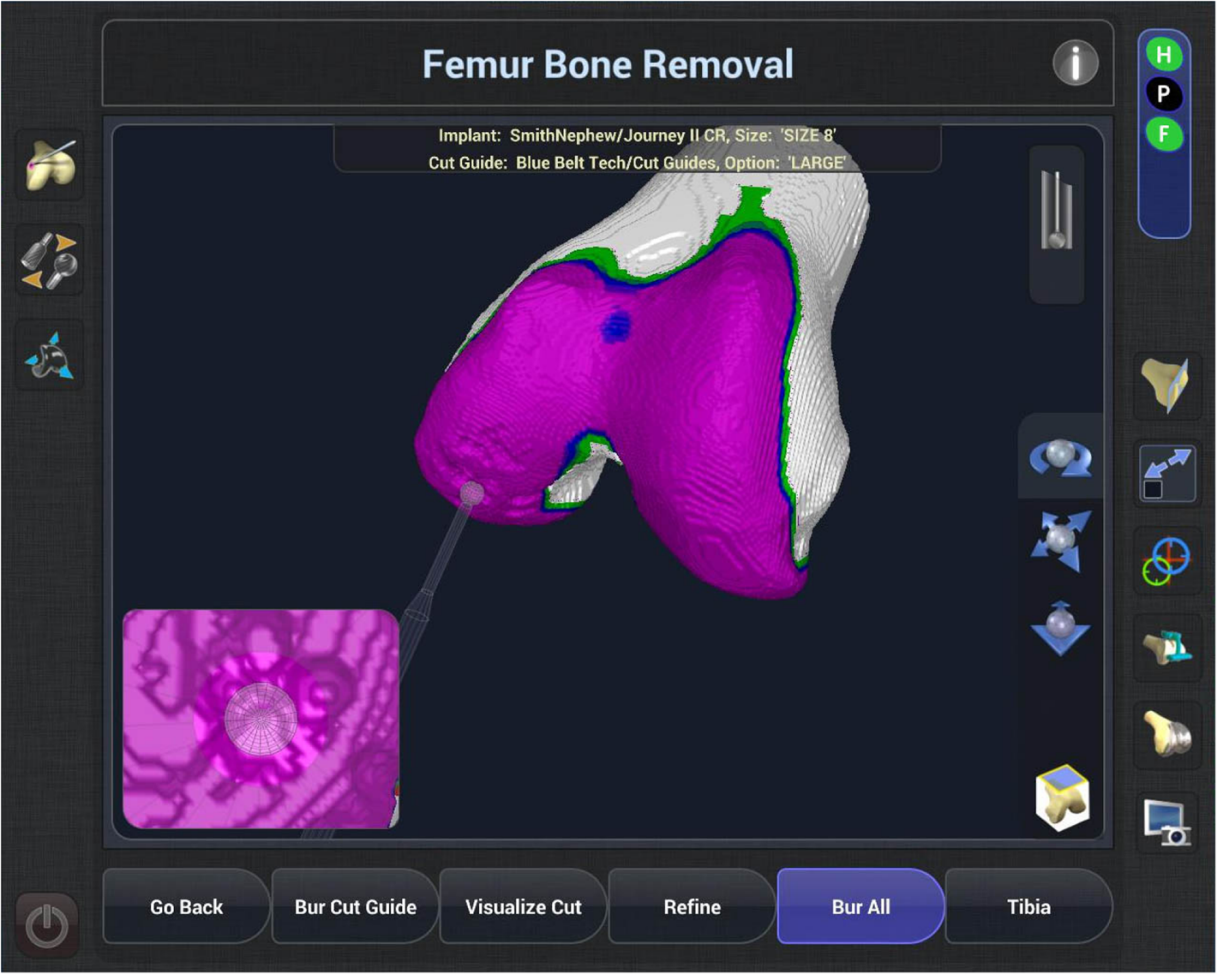
Screen shot from the NAVIO (Smith and Nephew) console of robotically-assisted TKA demonstrating the ‘planning page’. RTKA systems allow surgeons to modify implant position and size to achieve a desired plan. The planning page gives feedback to the surgeon about the effect of the adjustments on gap balancing

**Fig. 5:**
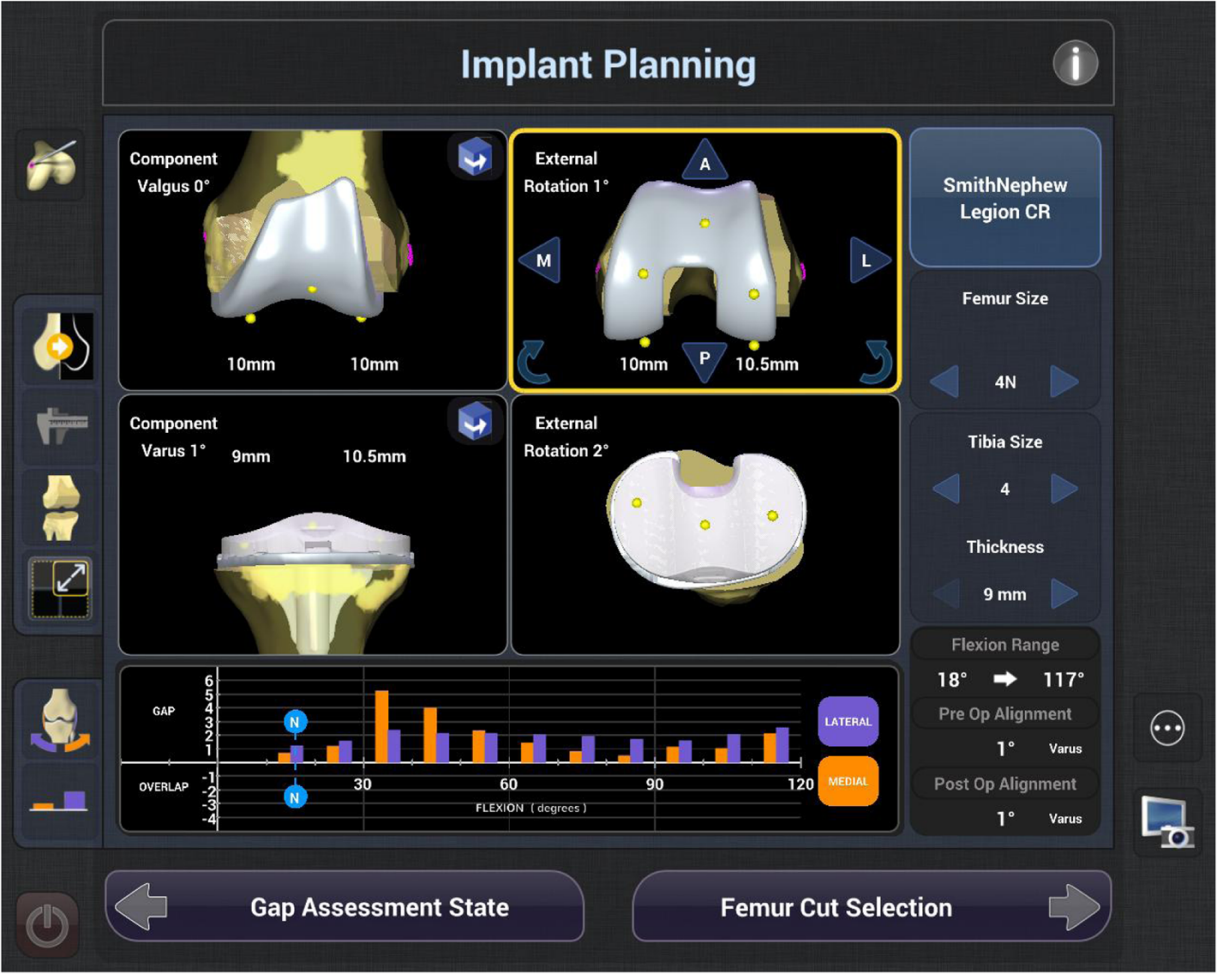
Screen shot from the NAVIO (Smith and Nephew) console of robotically-assisted TKA demonstrating planned bone resection (purple). Robotic-systems allow implant position and sizing to be ‘virtually’ trialled giving the surgeon feedback on the effect on gap balancing, notching, prosthesis overhang etc

Robotic TKA uses computer software to convert imaging into a virtual 3D reconstruction of the knee joint. This reconstruction can either be imageless, through anatomical landmarks gained intra-operatively such as the Navio Surgical system – Fig. 2 (Smith & Nephew, Andover, Texas), or image-based through pre-operative radiographs such as the Rosa Knee System (Zimmer Biomet, Warsaw, Indiana), CT such as the Mako Robotic Arm Interactive Orthopaedic System (Stryker Ltd., Kalamazoo, Michigan), or a combination of pre-operative imaging and intra-operative landmarks used to morph a model such as with the Omnibotic (OMNIlife Science Inc., East Taunton, Massachusetts). Once the virtual reconstruction is created, the surgeon plans the operation and gains feedback from the model regarding the effect of changing of parameters such as implant size, position or cut orientation, on balancing and overall alignment (see Figs. 3 and 4). The surgeon then uses the robotic arm to execute this plan. Robots also usually have boundary constraint, which avoids the cutting blade or burr going beyond the planned surgical field, in order to reduce soft tissue trauma (see Fig. 5).

## Results

### Accuracy of CAS TKA compared to conventional TKA

Alignment and implant accuracy outcomes have received the most attention in the literature. Nearly all studies show better accuracy of CAS TKA compared to conventional methods [16–28]. Interestingly, the benefit of navigation appears most consistent in the coronal plane. Fewer studies have reported accuracy of axial alignment, with just two studies showing improved rotational alignment for CAS TKA compared to conventional TKA [23, 29]. One possible explanation for this observation is that in some instances navigation may only be used for accuracy of coronal cuts, and sagittal and axial alignment in some systems are either not measured, or surgeons may prefer to use manual techniques for this part of the procedure, and this is not captured in the literature. In addition, for the more commonly used image -free systems, rotational landmarks require surgeon identification without any assistance from the computer, and landmarks such as epicondyles are less reproducible than simpler landmarks such as centre of femur and tibia.

Only one study comparing implant accuracy between RATKA and CAS TKA techniques has been published [30]. In a retrospective study of 81 matched patients, RATKA was 0.5 degrees closer to planned coronal alignment than CAS TKA when comparing the PRAXIM robot to a Stryker navigation system. In this study 37% of the femoral cuts were within a half degree of the planned cut angle, 63% of axial rotations were within a half degree, and 50% of the tibia slope cuts were within a half degree of the planned value.

### Patient reported outcomes for CAS TKA compared to conventional TKA

PROMs of CAS TKA compared to conventional TKA are summarised in Table 1. PROM data was available from 19 studies totalling 1978 TKA. 12 of the studies reported higher PROM scores compared to conventional TKA although this often did not reach statistical or clinical significance. Follow-up periods range from 2 to 15 years. None of the studies found a statistically significant difference between CAS TKA and conventional TKA PROMs at any time point, however there is a weak but consistent benefit for navigation over conventional TKA at short, medium and long-term follow-up.

**Table 1:** CAS TKA versus Conventional TKA PROMs

						Reported PROMs						
						Conventional TKA			CAS TKA			
Author	Year	Navigation	Implants	Follow-up	Study size							
Roberts [31]	2020	Stryker ASM/FNS	Triathalon	Mean 4.5 years	CAS = 90	OKS 41.59			OKS 41.74			
					CON = 78							
Selvanayagam [32]	2019	Orthopilot	Columbus CR – Braun	Mean 4 years	CAS = 25	WOMAC 16 KSS 82			WOMAC 16.5 KSS 81.5			KSS 82
					CON = 25							
Hsu [26]	2019	Not reported	DePuy Press fit Sigma	Mean 8.1 years	CON = 56	WOMAC 93.9		HSS 91.7	WOMAC 93.5		HSS 89.2	CON ROM 126 CAS ROM 127
					CAS = 56							
D’Amato [33]	2018	Stryker Imageless	Scopio/Optetrack PS	10 years	CON = 45	KOOS 78.6	KSS-F 87.4		KOOS 82.3	KSS-F 85.0		
					CAS = 48							
Petursson [34]	2018	Not reported	Not stated	2 years	CAS = 87	KOOS 88.2	KSS 69.7	EQ-5D 88	KOOS 85.6	KSS 69.2	EQ-5D 85.9	
					CO*N* = 80							
Kim [35]	2017	BrainLab	NextGen PS	15 years	CON = 141	WOMAC 14	KSS 92		WOMAC 15	KSS 93		CON ROM 128 CAS ROM 127
					CAS = 141							
Goh [36]	2017	BrainLab	Not stated	2 years	CON = 76	OKS 33.7	KSS 37.1	SF-36 physical 34.9	OKS 36.4	KSS 36.3	SF-36 physical 32.2	
					CAS = 38							
Ollivier [37]	2017	Not reported	Zimmer Next Gen	Mean 11 years	CAS = 40	KOOS 86.75	KSS 82	SF-12 physical 73	KOOS 85.5	KSS 82.5	SF-12 physical 72	
					CON = 40							
Todesca [38]	2016	Amplivision	Amplitude	Mean 6.4 years	CAS = 121	WOMAC 22	KSS 89	HSS 90	WOMAC 20	KSS 96	HSS 94	CON ROM 125
					CON = 117							CAS ROM 128
Baumbach [18]	2016	Orthopilot	Aesculap Search prosthesis	Mean 10 years	CON = 46	HSS 82.1	KSS 135.6	SF-36 physical 48.3	HSS 82.6	KSS 141.3	SF-36 physical 43.3	
					CAS = 50							
Song [39]	2016	Orthopilot	e.motion Braun	9 years	CAS = 37	WOMAC 9.9	KSS 82.2	HSS 88.2	WOMAC 11.2	KSS 83.8	HSS 91.9	
					CON = 38							
Ouanezar [40]	2016	Amplivision	Cementless mobile-bearing SCORE – Amplitude	10 years	CAS = 81		KSS 163			KS 164		CON ROM 114 CAS ROM 114
					CON = 57							
Cip [41]	2014	BrainLab	Next Gen Mobile bearing and PS Flex	Mean 5.5 years	CAS = 74	WOMAC 25.9	KSS 93.4	HSS 90.6	WOMAC 18.1	KSS 96	HSS 93.3	CON ROM 120
					CON = 79							
												CAS ROM 119
Lin [42]	2013	BrainLab	PS NextGen Zimmer	2 years	CAS = 30		KSS 29.6			KSS 29.4		CON ROM 128 CAS ROM 128
					CON = 35							
Thiengwittayaporn [43]	2013	Zimmer/Medtronic AxiEM electromagnetic navigation system	PS NextGen Zimmer	5 years	CAS = 58		KSS 84.5			KSS 85		CON ROM 121 CAS ROM 124
					CON = 58							
Lützner [44]	2013		Scorpio PCS	5 years	CAS = 34		KSS 150.2	Euroquol 61.7		KSS 149	Euroquol 66.8	
					CON = 33							
Tolk [45]	2012	Brainlab	LCS mobile bearinf	5 years	CAS = 50	OKS 39.4	KSS 155.6		OKS 39.1	KSS 156.4		CON ROM 112.8 CAS ROM 111.8
					CON = 50							
Harvie [46]	2012	Stryker	Duracon	Mean 5 years	CON = 22	WOMAC 21.84	KSS 148.1	SF-36 physical 65	WOMAC 15.97	KSS 157	SF-36 physical 45	
					CAS =24							
Hoppe [47]	2012	Orthosoft Navitrack	Innex UCOR TKA (Zimmer) with rotating platform	5 years	CAS = 28		KSS 94			KSS 94.6		CON ROM 117 CAS ROM 110
					CON = 28							
Seon [48]	2009	Orthopilot	e.motion	Not available	CAS = 43	WOMAC 70.9		HSS 67.2	WOMAC 70.9		HSS 67.2	CON ROM 120.2 CAS ROM 121.4
					CON = 42							
Kim [35]	2007	Brainlab	PFC -Sigma	Mean 2.3 years	CAS = 115		KSS 93	HSS 89		KSS 94	HSS 90	CON ROM 125 CAS ROM 127
					CON = 115							

*CAS* Computer-assisted TKA, *CON* Conventional TKA, *ROM* Range of motion, *KSS* Knee Society Score, *HSS* Hospital for Special Services Score, *SF-36* Short-form 36, *OKS* Oxford knee score, *WOMAC* Western Ontario and McMaster Universities osteoarthritis index

### Survivorship outcomes for CAS versus conventional TKA

Results are summarised in Table 2. US data [52] shows computer navigation increased in use from 1.2% in 2005 to 6.3% in 2014and 24,084 (0.4%) use robotic assistance for TKA. The proportion of technology-assisted TKAs has increased from 1.2% in 2005 to 7.0% in 2014. Computer navigation increased in use from 1.2% in 2005 to 6.3% in 2014. In Australia in 2002 2.4% (526) of all TKA were performed using CAS, and by 2018 this had increased to 33% (18,529) of all TKA [1]. In 2014, data on CAS TKA included 10 different navigation systems, but Brainlab (48.6%) and Stryker (31.8%) made up the majority of cases reported to the Australian registry. No survivorship data comparing robotically-assisted to CAS TKA has been published to date, however short-term data from the AOA ANJRR on robotic UKR has demonstrated reduced revision rates at 3 years compared to non-robotic UKR (2.8% versus 4.6%) [1].

**Table 2:** CAS TKA versus Conventional TKA Survivorship

					Survivorship	
Author	Year	Navigation	Implants	Follow-up	CAS TKA	CON TKA
Roberts [31]	2020	Stryker ANS/ASM	Triathalon	10 years	95.6% (*n* = 10,404)	95.1% (*n* = 9501)
					< 65 years 95.6%	< 65 years 95.1%
Selvanayagam [32]	2019	Orthopilot	Columbus CR - Braun	4 years	100% (*n* = 40)	100% (*n* = 40)
D’Amato [33]	2019	Stryker	Scorpio/Optitrak	10 years	96.2% (*n* = 48)	94.3% (*n* = 45)
Ollivier [37]	2017	Unknown	NextGen	13 years	97% (*n* = 40)	97% (*n* = 40)
Todesca [38]	2017	Amplivision	Amplitude	7 years	100% (*n* = 117)	100% (*n* = 121)
Kim [49]	2017	BrainLab	NextGen PS	15 years	99% (*n* = 141)	99% (*n* = 141)
Dyrhovden [50]	2016	Misc.^a^	Misc. ^b^	8 years	94.8% (*n* = 354)	94.9% (*n* = 2836)
					< 65 years 93.6% (*n* = 126)	< 65 years 92.4% (*n* = 955)
Baumbach [18]	2016	Not stated	Aesculap Search prosthesis	10 years	98% (*n* = 50)	87% (*n* = 46)
Ouanezar [40]	2016	Amplivision	Cementless mobile-bearing SCORE - Amplitude	10 years	91% (*n* = 87)	86% (*n* = 51)
Song [39]	2016	Orthopilot	e.motion Braun	9 years	100% (*n* = 37)	95.3% (*n* = 38)
De Steiger [51]	2015	Multiple	Multiple	9 years	95.4%	94.8%
					< 65 years 93.7%	< 65 years 92.2%
Cip [41]	2014	BrainLab	Next Gen Mobile bearing and PS Flex	5.5 years	98.9% (*n* = 74)	95.4% (*n* = 79)

^a^Prosthesis brands (AGC, Duracon, e.motion, LCS complete, and Profix)

^b^Navigation systems (Brainlab, Orthopilot, and Stryker)

*CAS* Computer-assisted TKA, *CON* Conventional TKA, *KSS* Knee Society Score, *HSS* Hospital for Special Services Score, *SF-36* Short-form 36, *OKS* Oxford knee score, *WOMAC* Western Ontario and McMaster Universities osteoarthritis index

The Australian National Joint Registry 2019 Annual report on 132,211 TKA performed using CAS showed a small benefit overall for revision rates compared to conventional TKA. When analysed for patients under 65 this benefit is more pronounced. Comparing CAS TKA to conventional TKA, revision rate at 15 years was 7.1% versus 7.4%. In patient over 65, revision rate was 5.4% for CAS TKA versus 5.2% for conventional TKA at 15 year follow-up. For patients under 65 for conventional TKA, revision rate was 9.7% versus 11.2% respectively. De Steiger [51] first reported this observation in 2015, noting that the rate of aseptic loosening was higher in the conventional TKA group. More recent data shows this trend continuing and becoming more pronounced in the under 65 group with time.

Data from 23,884 primary total knee replacements without patella resurfacing, reported to the Norwegian Arthroplasty Register during the years 2005–2014, were evaluated by Dyrhovden et al. [50]. Analysis of the 5 most used prosthesis brands (AGC, Duracon, e.motion, LCS complete, and Profix) and the 3 most frequently used navigation systems (Brainlab, Orthopilot, and Stryker) were reported. At 8 years followup, the revision rate was 5.1% in the CAS group and 4.2% in the conventional group. For people < 65, revision rate at 8 years was 6.4% for CAS versus 7.3% for conventional TKA.

Data from the New Zealand Joint Registry [31] compared outcomes in nearly 20,000 TKA performed by high volume arthroplasty surgeons (> 50 per annum) with mean 4.5 years follow-up. CAS was used in 10,404 TKA and conventional instrumentation was used in 8817. Data in this report was with a single CAS system and implant type (Triathlon TKA; Stryker Orthopaedics) using Stryker’s Full Navigation System (FNS) and its abbreviated variant, the Articular Surface Mounted (ASM) Navigation System. In patients < 65 years of age, the 5-year cumulative revision rate was 3.0% for the CAS group and 2.9% for the conventional TKA. At 10 years, the cumulative revision rates for patients < 65 years of age was 4.4% for the CAS group and 4.9% for the conventional TKA group and this difference was not deemed to reach statistical significance In comparison, data from the Australian registry at 10 year follow-up showed a revision rates in patients < 65 of 6.9% for CAS and 7.8% for conventional, showing a higher revision rate than was seen in the NZ data.

In a smaller study of 135 knees with 10 year follow-up, there was a trend towards a higher rate of revision in non-navigated TKA using an uncemented prosthesis. This was explained by increased rates of secondary patella resurfacing being required in the conventional group [40]. Kim et al. [49] demonstrated 98% survivorship in both conventional and CAS TKA in patients under 65% with minimum 14 years follow-up.

Whilst some of the larger registries report improved survival with the use of computer navigation for TKA, this finding is not universal across all registries and all studies examining this. It would certainly appear that the improved alignment achieved with CAS may have a beneficial effect on reducing the revision rate from wear and loosening, particularly in younger, more active patients, but ongoing analysis is clearly required before more definitive conclusions can be drawn.

### Accuracy of robotic TKA compared to conventional TKA

Data from 7 studies comparing implant accuracy of RATKA to conventional technique (Table 3) was available. RATKA has less outliers than conventional TKA. Whilst the trend towards improved accuracy is consistent amongst all studies, the strength of the difference between the two groups is heterogenous. Song et al. [57] reported no radiographic outliers in the coronal or sagittal alignment of RATKA, versus 20% coronal and up to 50% sagittal outlier inaccuracy of conventional techniques in a series comparing 100 TKA. Jeon et al. [55] also found improved implant accuracy of RATKA over conventional methods, but with a smaller difference (10.7% versus 16.5% coronal outliers for RATKA versus conventional). Interestingly, RATKA appears to improve implant accuracy by a similar difference in the coronal and sagittal planes compared to conventional TKA. There is currently insufficient comparative data regarding implant axial accuracy between these two groups.

**Table 3:** Studies Comparing Radiographic Outliers in RATKA Versus Conventional TKA

					Percentage of Radiographic Outliers			
Author	Year	Robot	Number	C-Mechanical %^a^	C-Femur	C-Tibia	S-Femur	S-Tibia
Yang [53]	2017	ROBODOC Vs Next Gen	113	R - 8.7	R - 5.8	R - 1.5	R – 14.	R – 8.7
				C - 33	C - 31	C - 10.3	C - 59	C - 41
Kim [54]	2019	ROBODOC Vs Duracon	1348	R - 14	R - 11	R – 11	R - 12	R - 11
				C - 26	C - 21	C – 20	C − 21	C - 20
Jeon [55]	2019	ROBODOC NextGen (Robotic)	163	R – 10.7	R - 8.3	R − 11.9	R – 3.6	R - 20.2
		Triathalon (conventional)		C - 16.5	C – 11.4	C - 11.4	C − 6.3	C − 15.2
Cho [56]	2018	ROBODOC	390	R – 10.6	R - 8	R – 7.1	R – 35.9	R – 5.3
				C - 26.4	C - 15	C – 7.9	C – 32.9	C – 32.1
Song [57]	2013	ROBODOC vs NextGen	100	R – 0	R – 0	R − 0	R – 0	R – 2
				C − 24	C - 4	C – 6	C- 0	C - 6
Song [58]	2011	ROBODOC vs NextGen	60	R – 0	R – 0	R – 0	R – 0	R – 6.7
				C – 23.3	C – 26.7	C – 0	C - 10	C - 50
Siebert [10]	2002	CASPAR Vs NextGen	120	R- 98	–	–	–	–
				C − 65				

*C* Coronal alignment, *S* Sagittal alignment

^a^Percentage of cases > 3 degrees from planned alignment or position

### Patient reported outcomes for robotic-assisted TKA compared to conventional TKA

Results are summarised in Table 4. As expected, data on RATKA is relatively small in numbers and shorter in follow-up when compared with either CAS or manual instrumented techniques. Ten-studies were found comparing PROMs between robotic and navigated TKA. Results show a small benefit to RATKA without reaching statistical significance in any of the studies reviewed. Eight of the ten compare the ROBODOC robot to conventional TKA, and the remaining 2 compare the MAKO robot. Interestingly, one study [59] found a 20% higher patient satisfaction rate in patients undergoing RATKA versus conventional despite no statistically significant difference in ROM, WOMAC or knee scores.

**Table 4:** Robotic TKA versus Conventional TKA PROMs

					Reported PROMs		RTKA
Author	Year	Robot	Follow-up	Study size	Conventional TKA		ROM
Smith [59]	2019	MAKO vs Triathalon	1 year	RTKA 120	Satisfied/very satisfied (Likert) 82%	Satisfied/very satisfied (Likert) 94%	RTKA 0–119
				CON 113		KSS Function - 80	CON 1–116
					KSS Function – 73		
Kim [54]	2019	ROBODOC Vs PS Duracon	13 years	RTKA 674	KSS-KS 93	KSS-KS 92	RTKA 125
				CON 674	WOMAC 18	WOMAC 19	CON 128
Jeon [55]	2019	Robodoc (NextGen) Vs Triathalon	10 years	RTKA 84	KSS 91.9	KSS 89.7	RTKA 137.2
				CON 79	KSS Function 85.4	KSS Function 89.5	CON 134.5
					SF-36 (physical) 47.2	SF-36 (physical) 47.5	
Cho [56]	2018	ROBODOC Vs NextGen	10 years	RTKA 160	HSS 86.7	HSS 88.5	RTKA130.7 CON 130.0
				CON 230	KSS Pain 45.8	KSS Pain 45.3	
					KSS Function 88.4	KSS Function 87.8	
					WOMAC 13.0	WOMAC 10.1	
					SF-36 P 47.6	SF-36 P 48.3	
Yang [53]	2017	ROBODOC vs NextGen	10 years	RTKA 71	HSS 88.7	HSS 87.2	RTKA 132.6 CON 131.0
				CON 42	WOMAC 11.5	WOMAC 7.6	
					VAS 1.1	VAS 1.2	
Marchand [60]	2017	MAKO Vs CR Triathalon	6 months	RTKA 20	Pain score 5/10	Pain score 3/10	
				CON 20	WOMAC 14	WOMAC 7	
Liow [61]	2016	ROBODOC vs NextGen	2 years	RTKA 31	OKS 17.7	OKS 18.3	RTKA 1.5–118.3
				CON 29	KSS-F 73.9	KSS – F 77	CON 1.7–125.2
					KSS-KS 87.9	KSS – KS 81.8	
					SF-36 Physical 66.9	SF-36 Physical 79.5	
					Satisfied (%) 89.7	Satisfied (%) 93.5	
Song [57]	2013	ROBODOC Vs NextGen	5.4 years	RTKA 50	WOMAC 30	WOMAC 28.9	RTKA 128
				CON 50	HSS 94.7	HSS 95.7	CON 129
Song [58]	2011	ROBODOC Vs CR NextGen	1.4 years	RTKA 30	WOMAC 13	WOMAC 11	RTKA 129
				CON 30	HSS 94.7	HSS 95.2	CON 129
Park [11]	2007	ROBODOC Vs Zimmer LPS	3.75 years	RTKA 30	KSS 90.4	KSS 91.6	RTKA 122
				CON 32	Knee Functional Score – 88.5	Knee Functional Score – 87.9	CON 118

*CAS* Computer-assisted TKA, *RTKA* Robotic TKA, *KSS* Knee Society Score, *HSS* Hospital for Special Services Score, *SF-36* Short-form 36, *OKS* Oxford knee score, *WOMAC* Western Ontario and McMaster Universities osteoarthritis index, *VAS* Visual Analogue Scale

### Robotic-assisted TKA current trends and survivorship compared to conventional TKA

Limited long-term data is available that compares robotically-assisted to conventional TKA, and owing to the short-term follow-up national joint registries have not yet made reports on robotically-assisted TKA publicly available. Results are summarised in Table 5. Only one study demonstrated improved implant survival comparing RATKA to conventional TKA [53], and no study demonstrated worse results with RATKA.

**Table 5:** Robotic TKA versus Conventional TKA Survivorship

					Survivorship	
Author	Year	Navigation	Implants	Follow-up	RATKA	CON TKA
Kim [54]	2020	ROBODOC	PS Duracon	15 years	100% (*n* = 724) 84	100% (*n* = 724) 74
Jeon [55]	2019	ROBODOC	NextGen (Robotic)	10 years	98.8% (*n* = 84)	97.5% (*n* = 79)
			Triathalon (conventional)			
Cho [56]	2019	ROBODOC	NextGen	13.5 years	98.8% (*n* = 160)	98.5% (*n* = 230)
Yang [53]	2017	ROBODOC	NextGen	10 years	97.1% (*n* = 71)	92.3% (*n* = 42)

*RATKA* Robotic-Assisted TKA, *CON* Conventional TKA

## Discussion

The most important finding from our review was that CAS TKA improves accuracy and consistency of implant position, and appears to provide a small improvement in PROMs and implant survival compared to conventional TKA. RATKA likewise improves implant accuracy compared to conventional techniques and early results suggest a similar small benefit in PROMs compared to conventional TKA. A strengthening trend is emerging showing CAS TKA has greatest benefit to implant survival in people under 65. RATKA survival analysis data is more limited and early results do not allow strong conclusions, however early trends are similar to CAS TKA.

The benefit of improved alignment accuracy appears slightly greater in the coronal plane compared to the sagittal plane. This is consistent with a recent meta-analysis showed improved accuracy when comparing CAS to conventional TKA [7]. In this study pooled results showed femoral component alignment was satisfactory in 95% of cases versus 84% of cases in the conventional group. Similarly with the tibial components, malalignment was present in 21% of conventional TKA versus 5% of CAS TKA. Fewer studies have reported accuracy and consistency with rotational alignment, but both CAS TKA and RATKA appear to be more accurate in the axial plane as well compared to RATKA [23, 29]. This is consistent with a previous metanalysis that found CAS TKA had fewer outliers than conventional TKA for rotational accuracy of implant position [62]. We did not identify any studies comparing axial accuracy of implant positioning of robotic to conventional TKA, although it would seem intuitive that image-based systems may allow more accurate identification of rotational landmarks, and this should be a focus of future research.

This clear difference in radiographic parameters is not consistently reflected in PROMs. Nonetheless, there does appear to be a small advantage to PROMs when using CAS over conventional TKA. These results are consistent with a recent meta-analysis of level 1 and 2 studies comparing CAS to conventional TKA [63], although it is worth noting that despite often finding significant differences, the minimal clinically important difference is often not reached [64]. It is not well established why improvements in accuracy do not lead to larger differences in PROMs, but there are several possible explanations for this. The majority of studies fail to measure rotational alignment accuracy, and this can certainly influence outcomes. It is also possible that small differences in coronal and sagittal alignment may not have a clinically meaningful impact on outcomes, or that current measurement tools are not sensitive to measure the differences in outcome that patients may otherwise appreciate. Other variables not controlled, particularly more recently, are those introduced by different alignment philosophies. Similar results were found when comparing PROMs for RATKA to conventional TKA, and whilst there was a trend towards improved outcomes for RATKA, no study found a statistically significant difference and these findings are similar to a recent meta-analysis by Ren et al. [65]. PROMs for RATKA are comparatively smaller in number and have shorter follow-up than navigated TKA, and it is therefore it is difficult at this point to draw any strong conclusions from the data available.

Survivorship shows promising signs for CAS TKA compared to conventional TKA, particularly in people under the age of 65. In our review, no study showed conventional TKA being superior to CAS TKA in survival analysis, and 5 [18, 39–41, 51] of 11 studies reported improved survival with CAS. It is plausible that malaligned implants causes eccentric polyethylene wear, with increased risk of aseptic loosening. This is supported by findings by Baumbach et al. who found at 10 year follow up an aseptic loosening rate of 17% of conventional and 9.8% of the navigated TKAs, with 58% versus 78% being within +/− 3 degrees of a neutral mechanical alignment [18]. However, Roberts et al. [31] reported New Zealand joint registry data and found no significant difference in survival for over or under 65 year old patients, when comparing CAS to conventional TKA, in contrast to Australian joint registry findings. It is important to note however, that this analysis only included outcomes of high-volume surgeons using a single prosthesis and navigation system (Stryker; Triathalon). Differences in registry results may also in part be due to prosthesis choice, such as the LCS which was reported to have inferior results with CAS in the short-term on the Norwegian registry [50] and the inability to account for other potentially important variables including surgeon volume and experience, and the type of navigation system used.

RTKA represents an extension of CAS systems, offering more comprehensive planning, additional feedback, and a precise delivery tool. Relatively limited data regarding survival analysis of RATKA to conventional TKA is available, but results show similar trends to that of CAS TKA [53–56]. Kim et al. [54] reported the longest follow-up data between conventional and RTA, and reported a remarkable 100% implant survival at 15 year follow-up for both conventional and RATKA groups. Yang et al. [53] reported 10 year outcomes of 113 TKA performed using the ROBODOC platform in comparison to conventional TKA. Cumulative survival in the robotic group and the conventional group was 97.1% and 92.3%, respectively, at 10 years. Both groups had 2 knees revised for infection. It should be noted that comparative studies to date have all used one robotic system, ROBODOC, and it unclear if other more contemporary robotic TKA systems will have different results. RATKA provides a more comprehensive surgical planning tool allowing for more precise implant positioning and sizing. It also provides virtual gap balancing, something that only some computer navigation systems provide. It is also important to note that most robotic systems must be used with a specific prosthesis, making this a potentially valuable marketing tool for the orthopaedic industry. Robotic systems also capture large volumes of data, which could potentially be used by industry to record and analyse surgeon preferences or techniques for future product development, and ownership and use of this data remains unclear.

Critics of CAS and RATKA site complication risk without evidence of clear benefit as reason for non-use. Pin secured navigation systems have in some studies been associated with tibial and femoral diaphyseal fractures in 1% of cases [66, 67] and a superficial wound infection rate of 1–5% [68, 69]. This has not been our experience. In the case of the senior author, over 4000 navigated TKA have been performed using pin fixation for navigation without sustaining a pin site fracture, and this complication can usually be avoided by not placing pins trans-cortically. Liow reported 16% vs 6.9% complication rate in RATKA vs conventional TKA, but operation length of time was similar. In this study, 3 out of 31 patients had RATKA aborted. However, Siebert et al. published the earliest series of RATKA using the CASPAR robot compared to the conventional technique with few complications in the RATKA group and a quick learning curve [10]. Another concern regarding CSA and RATKA is perceived increased length of operation time [24], and recent registry study from New Zealand [31] showed CAS TKA averaged 10 min longer operating time compared to conventional techniques. It is our experience that once surgeons become familiar with these techniques, additional operating time is negligible. There are of course additional practical considerations often not addressed in the literature. Surgical time is reported, but not set-up time which can be substantial in some cases. The requirement of additional industry support personnel to be present increases traffic in and out of theatre which may increase risk of periprosthetic joint infections, and robotic units require space in the operating room, which may already be overcrowded.

## Conclusion

Navigation in TKA was introduced with the intention of improving implant alignment with the hope that this would lead to improvement in PROMs and implant survival. Early data showed clear improvement in alignment, and early clinical outcomes showed promising results, with longer-term data and medium-term survival analysis recently emerging showing small benefits over conventional TKA. RATKA represents another phase of development, offering more comprehensive planning, additional feedback and a delivery tool. Early results show similar trends to that of CAS TKA with longer-term data still to come. These emerging technologies are tools available to surgeons, and surgeons need to be familiar with what is available, gain the appropriate experience necessary to use a system effectively, and decide which techniques provide them and their patients with the optimum outcomes. Further research is always necessary, and widespread adoption of new technology should always be evidence based.

## Supplementary information

**Additional file 1: Table 3.** Radiographic Accuracy of CAS TKA versus Conventional TKA

## Data Availability

All data and materials are contained in the manuscript or supplementary materials section.
