# Supplementary material for: Computer and robotic – assisted total knee arthroplasty: a review of outcomes
Source: J Exp Orthop. 2020 Sep 24;7:70. doi: 10.1186/s40634-020-00278-y (PMC7516005; doi:10.1186/s40634-020-00278-y)
Supplement: Supplementary file 1 — Additional file 1: Table 3. Radiographic Accuracy of CAS TKA versus Conventional TKA [file 40634_2020_278_MOESM1_ESM.docx]

| **Table 3. Radiographic Accuracy of CAS TKA versus Conventional TKA** | | | | | **Results** | | | |
| --- | --- | --- | --- | --- | --- | --- | --- | --- |
| Author | Year | Design | Navigation System | Comparison | Size | Coronal | Sagittal | Rotational |
| Baumbach | 2016 | Retrospective cohort | Orthopilot | Aesculap Search - instrumented | N=217 | Good – CAS 78%  CON 58% | - | - |
| Tolk | 2012 | Retrospective cohort | Brainlab | LCS mobile knee – De Puy | N=100 | Good – CAS 74%  CON – 50% |  |  |
| Lutzner | 2008 | Prospective RCT | Not stated | Scorpio | N=67 | Good – CAS 91%  CON – 68% | - | - |
| Kim | 2007 | Randomised control trial | Brainlab | PFC – Sigma | N=100 | Good – CAS 72%  CON – 65% | F – CAS 69%  F- CON 67%  T – CAS 75%  T – CON 91% | F – CAS 71%  F- CON 85%  T – CAS 45%  T – CON 51% |
| Chin | 2005 | RCT – CAS vs IM vs EM Measured using radiographic protocol | BrainLab - imageless | PFC Sigma EM and IM methods – De Puye | N=90 | Good*  CAS – 93.3%  EM – 73.4%  IM - 60% | Good**  CAS – 90%  EM – 63.3%  IM – 76.7% | Not measured |
| Bolognesi | 2005 | Retrospective single surgeon experience  Measured using long-leg films | Navitrack Orthososoft - imageless | Natural knee – Zimmer (IM femur/EM tibia) | N=100 | Good  CAS – 98% femur, 100% tibia  CON – 90% femur, 92% tibia | Not measured | Not measured |
| Jenny | 2005 | Multi-centre case-control | Orthopilot - imageless | IM femur, EM tibia  Prosthesis not reported | N=470 | Good – 92% CAS  72% - CON | F – Good – CAS 80%  Con 71%  T- good 85% CAS, 70% CON | Not measured |
| Anderson | 2005 | Case-control series | Stryker - imageless | IM femur, EM Tibia  PS SCORPIO | N=116 | Good – 95% CAS  84% - CON | F – 0-2flexion – 72% CAS, 63% CON  T – 2-5d 67% CAS, 55% CON | - |
| Bathis | 2004 | Prospective cohort | Brain lab - imageless | PFC Sigma implants with DePuye IM Femur and EM tibia guides | N=80 | Good – 96% CAS  78% - CON | - | - |
| Chauhan | 2006 | Randomised prospective trial | Stryker - imageless | Duracon implants, IM femur, EM tibia | N=70 | Less outliers, more consistency in achieving neutral mechanical alignment in CAS | F – no difference  T – improved in CAS | Femoro-tibial mismatch increased in CON vs CAS TKA |
| Decking | 2005 | Prospective RCT | Orthopilot | Condylar implants SEARCH Evolution prosthesis | N=52 | Good – CAS 96%  CON – 68% | F – CAS 74%  Con – 60%  T- CAS 74%  CON - 44% | - |
| Haaker | 2005 | Prospective matched cohort | Orthopilot | SEARCH Aesculap | N=200 | Good – CAS 79%  CON – 28% | Good – CAS 79%  CON – 28% | No difference |
| Dutton | 2008 | Prospective randomised study | BrainLab | PFC PS – De Puye Instrumentation | N=108 | Good – CAS 92%  CON 68% | T – CAS 0 to 7 in 90%  61% CON  F – not reported |  |
| Ensini | 2007 | Prospective randomised control trial |  |  | N=120 | Good – CAS 98.3%  CON – 80% | F – CAS 1.1 mean flexion  F – CON 2.8 mean flexion | Mean .1 degree IR CAS  Mean .9 degree IR CON |
| Hernández-Vaquero | 2010 | Prospective case-series | Stryker - imageless | - | N=80 | Good – CAS 90%  CON – 50% | - | - |

IM = intra-medullary

EM = extra-medulary

Saggital - +indicates posterior slope was increased by this amount

* Good coronal alignment - Within +/- 3 degrees perpendicular to mechanical axis

** Good saggital alignment – tibial component posterior slope within 2-4 degrees and femoral component within 0-3 degrees flexion

F = Femur

T= Tibia
